# Aging and Curing Temperature Effects on Compressive Strength of Mortar Containing Lime Stone Quarry Dust and Industrial Granite Sludge

**DOI:** 10.3390/ma10060642

**Published:** 2017-06-11

**Authors:** Muhammad Nasir Amin, Kaffayatullah Khan, Muhammad Umair Saleem, Nauman Khurram, Muhammad Umar Khan Niazi

**Affiliations:** Department of Civil and Environmental Engineering, College of Engineering, King Faisal University (KFU), P.O. Box 380, Al-Hofuf, Al-Ahsa 31982, Saudi Arabia; kkhan@kfu.edu.sa (K.K.); mmsaleem@kfu.edu.sa (M.U.S.); nrauf@kfu.edu.sa (N.K.); umar.niazi@nu.edu.com (M.U.K.N.)

**Keywords:** compressive strength, mortar, temperature, quarry dust, granite sludge

## Abstract

In this study, the researchers investigated the potential use of locally available waste materials from the lime stone quarry and the granite industry as a partial replacement of cement. Quarry sites and granite industry in the eastern province of Saudi Arabia produces tons of powder wastes in the form of quarry dust (QD) and granite sludge (GS), respectively, causing serious environmental problems along with frequent dust storms in the area. According to ASTM C109, identical 50-mm^3^ specimens were cast throughout this study to evaluate the compressive strength development of mortars (7, 28 and 91 days) containing these waste materials. Experimental variables included different percentage replacement of cement with waste materials (GS, QD), fineness of GS, various curing temperatures (20, 40 and 60 °C as local normal and hot environmental temperatures) and curing moisture (continuously moist and partially moist followed by air curing). Finally, the results of mortar containing waste materials were compared to corresponding results of control mortar (CM) and mortar containing fly ash (FA). The test results indicated that under normal curing (20 °C, moist cured), the compressive strength of mortar containing the different percentage of waste materials (QD, GS, FA and their combinations) remained lower than that of CM at all ages. However, the compressive strength of mortar containing waste materials slightly increased with increased fineness of GS and significantly increased under high curing temperatures. It was recommended that more fineness of GS be achieved to use its high percentage replacement with cement (30% or more) incorporating local environmental conditions.

## 1. Introduction

Most of the developed and underdeveloped countries are experiencing the serious problems of dumping non-biodegradable and industrial wastes. Dirty landfills, high transportation cost and environmental issues of waste materials have forced worldwide researchers to explore appropriate green solutions for dealing with such wastes. Furthermore, the issue of depletion and overexploitation of available natural resources has alerted the construction industry to look for alternatives. The building construction industry consumes a huge amount of naturally available materials and is responsible for about 24% of the total materials mined [1]. Concrete is the most widely-used material for building construction all over the world. In 2006 alone, the annual global production of concrete was around 25 billion tons [2]. According to the U.S. Geological survey report, about 4.1 billion tons of Portland cement were produced in 2015 [3]. The production of cement, which is the main binder in concrete, require a huge amount of natural material and energy. For the production of each ton of cement, approximately 1.5 tons of raw material is required [4]. The cement industry is also contributing around 5% of global CO_2_ emissions [5].

Much research has been done by many researchers to utilize the waste produced by different industries as a cement and aggregate replacement to minimize the environmental and ecological issues caused by cement and concrete industry. Substitution of waste materials will control the damages caused by the quarrying of raw material for making cement and concrete. The waste materials, such as fly ash, slag, silica fume, rice husk ash, ornamental stone waste and waste glass sludge, had been successfully used as partial replacement of cement [6,7,8,9,10,11]. The aggregate produced by using the waste material, such as fly ash, slag, palm oil shell, stone cutting sludge and demolition waste, has the ability to partially replace the normal aggregate or filler in concrete [12,13,14,15,16,17]. The potential use of these industrial wastes in the cement and concrete industry conserves natural resources, solves waste management issues, saves energy and reduces the amount of clinker and associated CO_2_ emissions.

More specifically, a huge amount of waste is being produced worldwide from aggregate quarry sites in the form of fine dust, mainly from crushing and washing processes of limestone aggregates’ crushing plants. As these materials are extracted from the ground and during this process, a significant amount of crushed dust and fine particles is transmitted to the surrounding air. Those quarry sites and crushing plants that exist in the vicinity of urban areas give rise to severe environmental-related issues and health hazards for the adjacent communities. Moreover, these figures become alarming during the process of crushing in terms of handling and transporting it to far, vacant places. An appropriate use of such wastes in the construction industry is always a useful step forward in many aspects such as protecting the environment, minimizing depletion of natural resources (less cutting of stones, less cement production), economy and many others. In addition to their deposits at the site, some of this fine dust also gets stuck to the crushed aggregate surfaces, leading to poor aggregate-mortar paste bonds. Therefore, in order to ensure a proper aggregate-mortar paste bond, this excessive dust is removed by washing aggregates at ready mixed concrete plants. Consequently, the wastes obtained in the form of sludge require dumping and treatment for disposal purposes.

By virtue of the relation with the construction industry, many researchers came forward to use such fine waste materials either in the form of filler or a cement replacer to address the issue of dumping wastes, as well as high CO_2_ emissions during the manufacturing of cement. In addition to quarry sites and aggregate washing wastes, the Kingdom of Saudi Arabia also retains rich deposits of ornamental stones [18]. The availability of these materials in abundance and at lower cost has motivated many to invest in the sector of granite and marble stone. This resulted in an increased activity of cutting, crushing, washing and polishing of these materials. All of these processes produce huge wastes in the form of dust and sludge causing not only dumping issues, but also threatening the surrounding environment. The best way to get rid of these wastes (quarry dust and granite sludge) is to use them as a raw material in the concrete and construction industry.

Among the inventory of secondary raw materials used as fillers and binders, the quarry dust (QD) and granite and limestone sludge were found to be the most popular of these. Galetakis et al. [19] reported that about 55% of researchers have investigated the potential use of QD and 31% examined the use of granite and limestone sludge, while 14% of the remaining research was focused on investigating the combined effect of QD and ornamental stone waste as a fine aggregate or cement replacement.

Past research works show that limestone QD has the potential to substitute ordinary Portland cement (OPC) up to a certain level [20]. The limestone QD normally acts as a filler, accelerates the early age hydration and forms a calcium carbo aluminate [21,22,23]. It has been reported that a 15% replacement of limestone QD with cement exhibited high early age strength due to the improved particle packing, high rate of cement hydration and its interaction with aluminate hydrates to form calcium carbo aluminate [24,25,26,27,28]. A similar strength behavior was reported when limestone QD was replaced with cement by 10% [29]. In addition to the replacement levels, many researchers also reported the importance of the fineness of limestone QD when used as a cement replacement. Hawkins et al. [30] suggested that limestone QD should be finer than cement in order to achieve a comparable result even at 8% replacement. However, Voglis et al. [26] reported that 15% replacement of limestone QD having blain fineness more than cement shows more strength at early ages up to 14 days and a comparable strength at 28 days to that of the control (100% cement) and other pozzolans. Limestone QD coarser than cement at a replacement level of 15% and 30% offered reduced strength at all ages and also showed poor durability performance [31]. Kumar et al. [32] showed that a limestone QD having an average particle size of 3 microns, replaced by 10%, gave strength close to that of control samples.

Like limestone QD, many researchers also evaluated the potential use of granite sludge (GS) as a replacement of cement in mortar and concrete. Marmol et al. [33] found that GS having a particle size less than 100 microns possesses comparative strength values at early ages (seven and 28 days) when replaced by 5% and 10% by mass of cement. Hamaiedeh et al. [34] also found that a 10% of GS replacement by cement gave nearly equal results to that of control samples, but higher substitution reduced the strength. However, according to Fairfield et al. [35], even a high percentage replacement of cement (such as a 30%) with powdered GS of a mean particle size of 45 microns attained strength close to the control sample at early ages (28 days) and lower later age (56 and 91 days) strengths which is due to its less pozzolanic potential at later ages. Contrary to the above researchers [33,34,35], Ramos et al. [36] proved that even a 10% substitution of cement with both coarse and fine GS produced lesser strength than corresponding control samples even at 28 days. However, they further demonstrated that GS exhibited better strength behavior with increased fineness without compromising workability and greatly enhanced the durability in terms of resistance to chloride ion penetration and alkali-silica reaction. Moreover, Bacarji et al. [37] also found that both marble and GS when mixed showed negligible pozzolanic activity. According to their results, a 5% substitution of cement showed reasonable strength while higher substitutions led to a reduction in strength due to its non-reactive nature. The less pozzolanic potential of GS was due to the presence of a much lesser amount of reactive SiO_2_ (22%) [38]. The pozzolanicity of granite powder was reported as low at early ages, but increases with the passage of time; and at 91 days, it exhibited similar potential as that of copper slag, but lower than other conventional pozzolanic materials, such as fly ash (FA) and silica fume [38].

In this study, pozzolanic potential and the compressive strength of mortar containing different percentages of locally available wastes (GS of different fineness and QD) were investigated under normal, as well as high curing temperatures. ASTM C618 [39] and ASTM C109 [40] were used to evaluate the pozzolanic potential and the compressive strength of mortar, respectively. The effects of different curing temperatures were not considered in previous studies; however, this could affect the development of the compressive strength of mortars due to its direct effects on the reaction rate. In general, high temperature variation occurs due to seasonal changes and the heat of cement hydration. Different curing temperatures (20, 40 and 60 °C) in this study were taken considering the local normal and hot environmental temperature conditions of most eastern and western parts of Saudi Arabia. Mortars containing FA were also cast in order to compare the results of this study with a very well-known pozzolanic material. With respect to mix proportions, various experimental variables include different percentage replacement of cement with GS (10% and 20%), QD (10% and 15%) and FA (10% and 20%). The different fineness of GS (100% passing 45 microns and 25 microns) and the mixes in combinations (FA + GS (20%), QD + GS (20%)) were also studied. Finally, all of the compressive strength results of mortar containing GS and QD were presented in this study and compared with the corresponding results of CM (100% cement) and the mortar containing FA.

## 2. Materials and Methods

### 2.1. Materials

ASTM C150 Type-I Portland cement obtained from a Saudi cement factory was used as the main binder material in this study [41]. A commercially available standard sand (EN 196-1 and ISO 679: 2009) recommended in ASTM C109 was used as a fine aggregate. Table 1 shows the sieve analysis results of sand. The fineness modulus (FM) of sand equal to 2.54 was calculated according to ASTM C125 [42]. The physical and chemical properties of the cement and all other cement replacing materials used in this study (QD, GS and FA) are given in Table 2. A brief description of the cement replacing materials used in this study is given below.

#### 2.1.1. Quarry Dust

**Figure 1:**
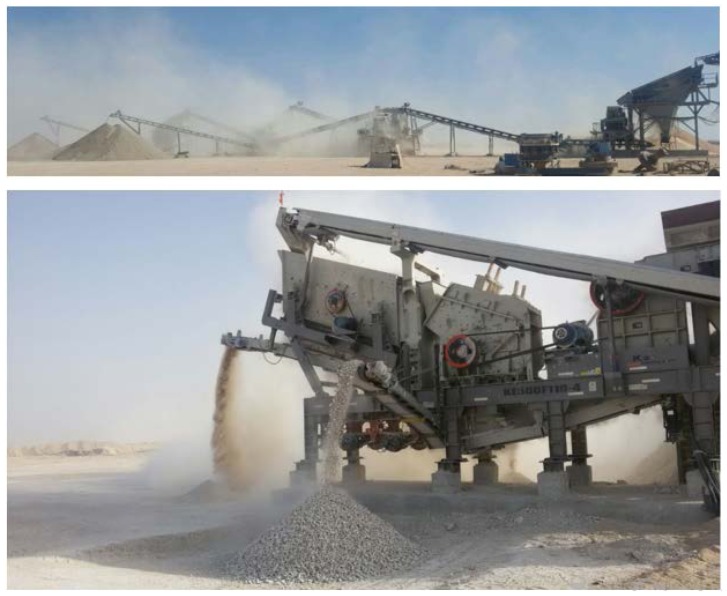
Generation of lime stone quarry dust (QD) at a quarry site located in the Al-Ahsa region, eastern province of Saudi Arabia.

The QD used in this study was directly obtained from the Al-Mousa quarry site (Figure 1) located in Al-Ahsa, the eastern province of Saudi Arabia (25°15′57.863″N, 49°8′44.08″E). QD is a byproduct of the aggregate industry formed during the crushing and washing stages of aggregate production. Limestone and dolomite produce more dust as compared to granite and basaltic rock. The central and eastern parts of Saudi Arabia consist of a sedimentary layer of younger geological age. Solid rock mainly consists of limestone and dolomite. In the central region, the limestone is dense and hard, and as it goes down towards the east, the rock becomes porous and soft. As a result of this, soft limestone rock available in the eastern region yields more dust as compared to the hard rock of the central region. A huge quantity of aggregate is produced for utilization in concrete production and road construction in Hofuf, Dammam and Jubail; an area of the eastern region of Saudi Arabia [43]. Therefore, during the mining, transporting and crushing processes for the production of aggregate, an enormous quantity of limestone powder is produced. During sandstorms, these fine particles cause severe environmental and health hazard issues. Therefore, there is always a vital need to properly utilize this waste for beneficial purposes such as in construction.

#### 2.1.2. Granite Sludge

**Figure 2:**
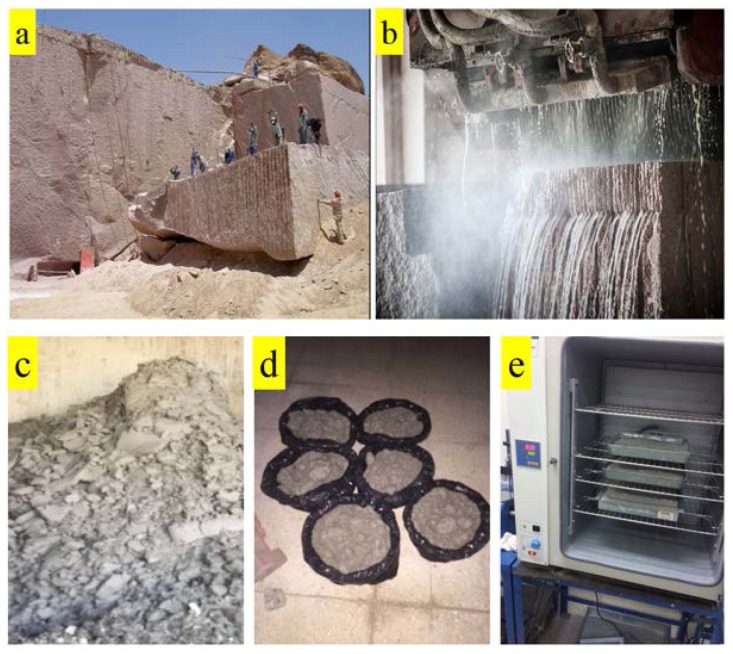
(**a**) Granite quarry site; (**b**) stone cutting process at the Saudi Marble and Granite Factory Co., Ltd.; (**c**) GS from washing, cutting and polishing; (**d**) air drying of GS for one week; (**e**) further drying in an oven at a standard temperature of 110 °C for 12 h.

This is the waste generated during sawing and surface finishing processes of granite used as an ornamental stone. A huge amount of waste is generated from the ornamental stone quarries available in Saudi Arabia [44]. Granite stone waste used in this study was collected directly from the Saudi Marble and Granite Factory Co., Ltd. (Riyadh, Saudi Arabia), which was one of the largest producers of granite products in the Middle East [45]. Figure 2a shows the granite quarry site, and Figure 2b shows the cutting of granite at the Saudi Marble and Granite Factory. The sludge waste as produced was collected directly from the Saudi Marble and Granite Factory (Figure 2c) and kept in air for one week (Figure 2d) followed by oven drying (Figure 2e) at 110 °C for 12 h to ensure that it was completely dried. Finally, the GS of different fineness was obtained by using Sieve# 325 of mesh size 45 microns (GS45) and Sieve# 500 of mesh size 25 microns (GS25).

#### 2.1.3. Fly Ash

In this study, a commercially available ASTM Type F fly ash was also used to compare the results of the current study with corresponding control mortar (100% cement) and mortar containing local waste materials such as GS and QD. Fly ash is a waste obtained as a byproduct of coal-fired power plants and a popular pozzolanic material being used all over the world [46]. The estimated global production of fly ash is around 500 million tons [5].

### 2.2. Particle Size Analyses, Scanning Electron Microscopy and X-ray Diffraction of Materials

Particle size analyses were carried out by laser diffraction using Microtrac S3500 (Microtrac Inc., Montgomeryville, PA, USA, complying with ISO 13320 [47]) with the turbotrac accessory. The d_10_, d_50_ and d_90_ sizes were calculated and listed in Table 3 to compare the particle size distribution of all materials used in this study. The particle size analyses’ results show that the both GS45 and GS25 are finer than cement, as well as fly ash; while, on the other hand, the limestone QD particles were coarser than cement particles. Along with particle size calculations, the Microtrac calculated specific surface area (CS in m^2^/cc) was also calculated for all materials as given in Table 2. A higher CS value means more blain fineness and vice versa.

**Figure 3:**
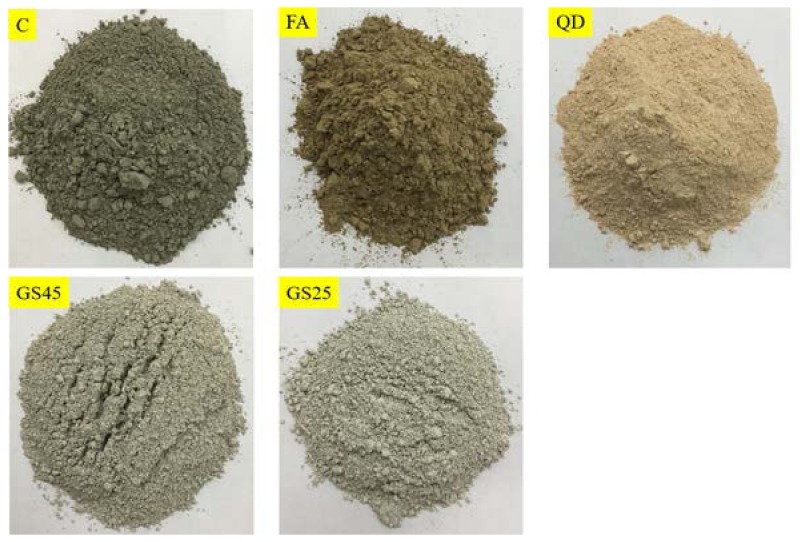
Materials in powder form as used in this study: cement (C); fly ash (FA); quarry dust (QD); granite sludge passing a 45-micron sieve (GS45) and a 25-micron sieve (GS25).

The value of fineness can be obtained in cm^2^/g by multiplying the Microtrac CS value by 10^4^ and then dividing by the density of the material (3.15 g/cm^3^ for cement). The correlation with Microtrac values allows easy conversion to Blaine and Wagner values and displayed using Microtrac FLEX software (Microtrac Inc., Montgomeryville, PA, USA). Using this software, the Blain surface area of OPC was determined as 360 m^2^/kg, which is almost equal to directly obtained Blain fineness of cement (Table 2). This authenticates the reliability of using the CS value as an important parameter for comparing the fineness of different materials. Figure 3 shows the materials in their final powdered form as used in this study.

**Figure 4:**
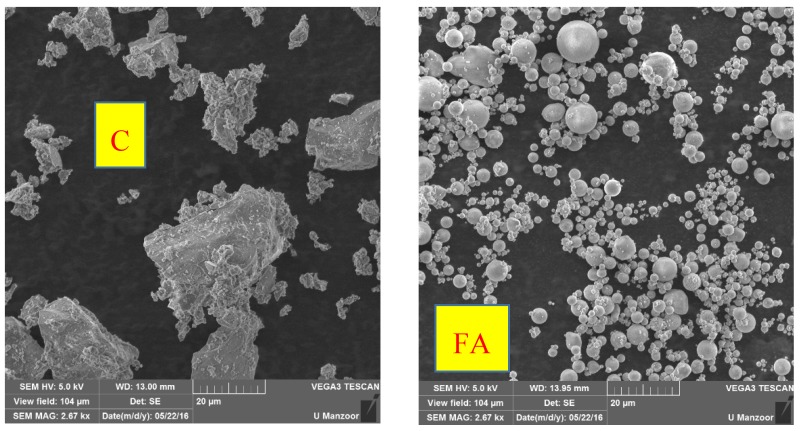

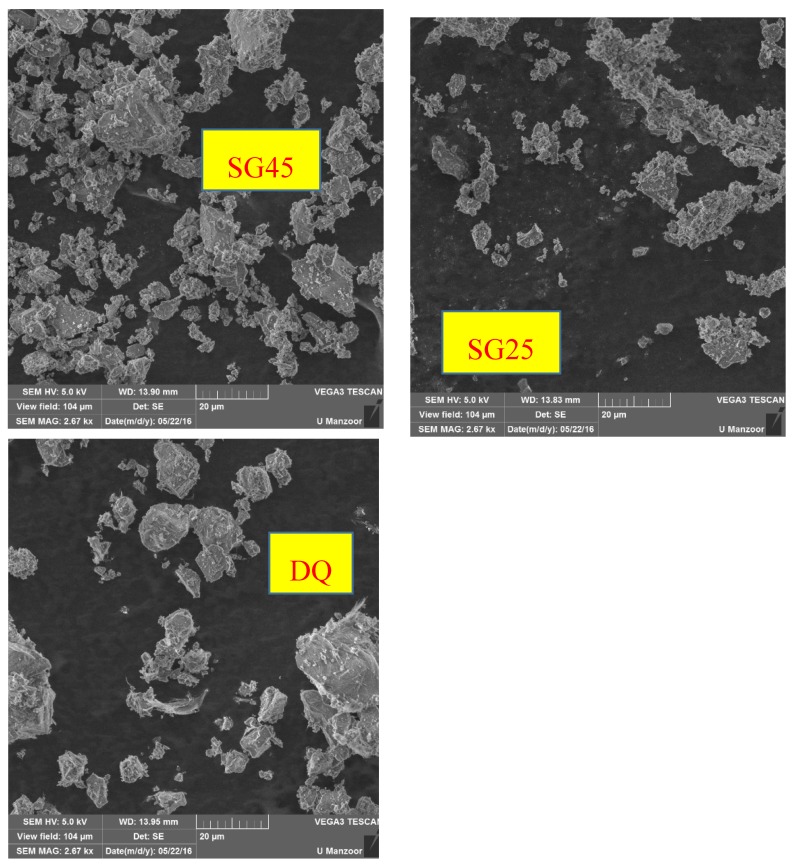
SEM images of materials: cement (C); fly ash (FA) having regular spherical particles; quarry dust irregular particles (QD); and granite sludge irregularly-shaped porous particles (GS45 and GS25).

In addition to particle size analyses, scanning electron microscopy (SEM) of materials in powder form (Figure 3) was also carried out using VEGA3 TESCAN (TESCAN, Brno, Czech Republic). The purpose was to study the morphology of these materials and to qualitatively authenticate the particle size analyses’ results (Figure 4). To perform SEM, beam energy and working distance were set at 5 kV and 13 mm, respectively. The SEM pictures (view field: 104 μm and scale: 20 μm) show that both GS and limestone QD particles are comparatively more angular, irregular, elongated and porous compared to those of cement. As usual, the shape of fly ash particles was found to be more uniform and spherical.

**Figure 5:**
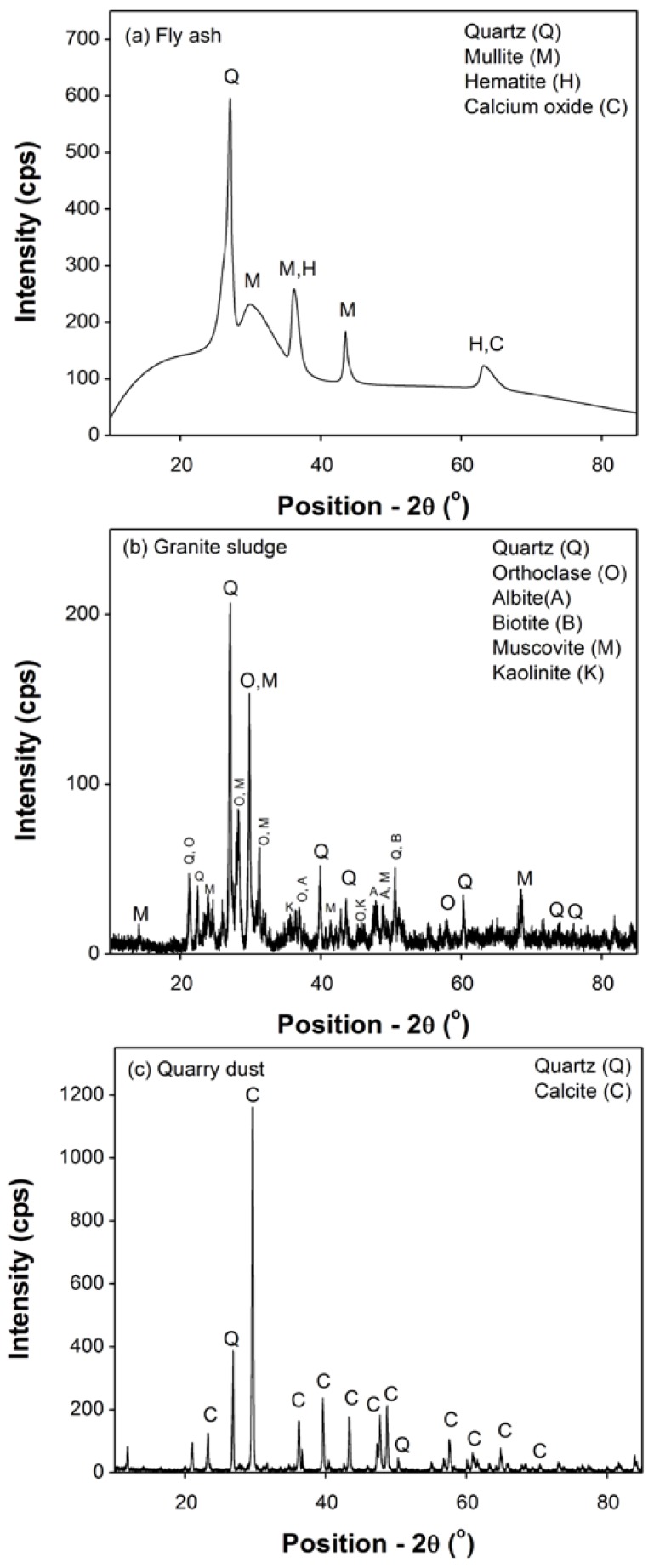
X-ray diffraction analyses of powdered materials fly ash (**a**), granite sludge (**b**) and quarry dust (**c**).

Moreover, the X-ray diffraction (XRD) analysis was also carried out to determine the different phases present in the materials. Specifically, this analysis was required to better understand the characteristics of the materials (crystalline/pozzolanic) and their influence on measured strength values. Figure 5 shows the XRD results of powdered samples of fly ash, granite sludge and quarry dust. The XRD patterns presented in Figure 5 were recorded by using Rigaku MiniFlex II (Rigaku, The Woodlands, TX, USA) at 10 and 85°. The radiation level for Cu Kα radiation was kept 40 kV and 40 mA, and the step size of 0.01 was maintained throughout the tests.

### 2.3. Mix Proportions and Test Methods

#### 2.3.1. Mix Proportions

As shown in Table 4, a total of eleven different mortar mix proportions were used in this study including the control mortar mix (CM). All of the mix proportions were different from one another in terms of the type of cement replacing materials (GS, QD, FA), different percentage replacement levels for each material (10%, 15% and 20%) and different fineness, such as GS passing 45 microns and 25 microns. For each mix proportion, the quantities of mix ingredients were calculated per batch to cast nine 50 mm^3^ specimens. It is worthy of mention here that the FA was used in this study just to compare its results with those of corresponding mortars containing GS and QD. The purpose of selecting the last two ternary mortar mixes (FA10 + GS25-10 and QD10 + GS45-10) was to investigate their influence on strength development when used in different combinations. The purpose of using ternary mixes was to gain the maximum benefit out of this work and find the best possible cement replacement combination between FA, GS and QD. Using GS alone or QD alone in higher percentages (up to 20%) may not produce strength as comparable to CM. In addition, using GS and QD alone would also lead to less workability, which can be enhanced by using FA with them due to its spherical particle shapes (Figure 4). According to ASTM C109 [40], the water to cementitious materials ratio (w/cm = 0.485) and the sand to cement ratio (s/c = 2.75) were kept constant throughout this study.

#### 2.3.2. Mortar Mixing

A three-speed Hobart mixer was used to mix the ingredients of mortar in accordance with the ASTM C305 [48]. At first, water and cement were poured into the mixing bowl, and the mixing was continued for 30 s at slow speed mode, followed by adding sand and mixing in the same speed mode for the next 30 s. After that, the mixer was stopped just to change its speed mode from slow to intermediate level and started mixing again for 30 s more. After 90 s of continuous mixing, the mixture was stopped for the next 90 s, and mixing started again, which finally lasted the next 60 s. The total time of mixing was maintained at 4 min, and the same steps were followed for each batch of mixing.

#### 2.3.3. Casting and Specimen Preparation

Immediately after mixing, the fresh mortar was cast into 3-gange 50 mm^3^ steel molds. Molds were filled in two equal layers. Rodding was done after filling each layer as specified in ASTM C109. For each mix (Table 4), in total, 27 specimens were cast to test compressive strength at three different ages (3, 7 and 28 days) and three different continuously moist curing temperatures (CMCT), such as 20, 40 and 60 °C. Considering the capacity of the Hobart mixer used, mixing was carried out in three batches for each mix, and the resulting fresh mortar was enough to cast nine 50 mm^3^ specimens. After casting, the molds were covered by a double-layered polyethylene sheet to prevent moisture loss from the top surface of the specimens and then kept at controlled room temperature (20 °C) for the next 24 h. After de-molding, all of the specimens were continuously moist-cured under different curing temperatures (20, 40 and 60 °C) until the age of testing. An additional 18 specimens were also cast for each of three selected mix proportions (CM, FA20, GS25-20) to further investigate the influence of the time of moist curing on strength development with respect to curing temperatures (20, 40 and 60 °C) at 28 and 91 days. These specimens were moist cured for first 7 days (7DM) followed by curing in the air of relative humidity 60% under the respective curing temperature until the age of testing.

#### 2.3.4. Testing

The compressive strength tests on mortar cubes were performed according to ASTM C109 [40]. A displacement control QUALITEST QM-300 (Qualitest Inc., Charlotte, NC, USA) universal testing machine (UTM) of capacity in compression 300 kN was used to perform the compression tests throughout this study. To avoid testing errors, every time, the new specimen was carefully placed in the testing machine exactly below the center of the upper bearing block. According to the standard test procedure of ASTM C109, a pacing rate of 1 mm/min was applied on specimens such that the maximum load was reached in neither less than 20, nor more than 80 s from the start of loading. The load data were logged automatically through a computerized data acquisition system linked to the UTM, and then, the ultimate strengths were calculated by dividing the load value with the bearing area of the tested specimen (2500 mm^2^). The test results of three identical specimens were averaged.

## 3. Results and Discussions

### 3.1. Characteristics of GS and QD

The pozzolanic potential of GS was evaluated by comparing with different classes (N, F and C) of ASTM C618 [39]. It was found that the GS meets the pozzolanic criteria of all classes of ASTM C618. For instance, the sum of SiO_2_, Al_2_O_3_ and Fe_2_O_3_ is 84.65%, which is more than the min requirement of 70%; the amount of SO_3_ is 0.10, which is less than the max limit of 4.0; and the loss on ignition (LOI) is 2.71, which is less than the max limit of 10.0 (Table 2). However, only 22% weight of the total silica (65%) present in GS was found to be reactive, which is very close to the minimum requirement level (25%) for pozzolanic materials [38]. The above results demonstrated that the GS can be used as a partial replacement of cement in concrete productions. Unlike GS, the chemical composition of QD shows that it mainly consists of CaC (49.8%) and may be treated just as a filler rather than a pozzolanic material. The chemical analysis was performed according to ASTM C25-11e2, and a low percentage of CaO was found, which could be due to the high LOI of 44.6% [49].

As mentioned earlier, XRD analyses of materials used in this study were also carried out, and the results are presented in Figure 5 to determine the crystalline phases present and learn about the pozzolanic phases present in this analysis. From the XRD analysis of fly ash (Figure 5a), it was found that it was mainly composed of quartz and glassy phases of aluminosilicate also known as the mullite phase. The glassy phase of mullite is distinguishable from the humpy nature of the XRD pattern, especially, at lower 2ϴ values. The quartz maximum intensity X-ray reflection was positioned at the 2ϴ value of 27.6 and was related to the (012) α-quartz plane, which can be used to calculate the average particle size. The result indicated the very fine nature of the quartz crystals in the fly ash. Therefore, due to its small size, the surface energy of quartz is expected to be very high, and it would react readily and show high pozzolanic reactivity in mortar matrix. On the other hand, the mullite phase would remain non-reactive during pozzolanic reactions [50]. Besides these two major phases, two additional phases, namely hematite and calcium oxide, were also found and discernible from the fly-ash XRD pattern.

As shown in Figure 5b, the XRD pattern of granite sludge showed the presence of multiple phases, including quartz, feldspar and phyllosilicate. The alpha-quartz gave typical XRD reflections at different 2θ values characterized by small peak-widths showing a larger particle size in Scherer’s analysis. Thus, the expected pozzolanic activity will be lower in the granite sludge. Apart from quartz, there were two distinguishable phases of feldspar; orthoclase and albite. Additionally, a phase of biotite, muscovite and kaolinite, a type of phyllosilicate, was also observed. The remaining smaller XRD signatures may correspond to different by-products present in smaller quantities. Nevertheless, the pozzolanic activity of granite sludge should be smaller due to its high crystallinity as compared to fly ash [38]. The XRD characterization of quarry dust showed sharp and intense reflections of the calcite phase (Figure 5c). The sharp and narrow reflection of quartz was also observed at 2θ values of 27.1 and 50.5. Thus, the low reactivity of the quarry dust can be attributed to its low surface activity and larger dust particle sizes.

In addition to the criteria related to chemical composition and XRD analysis, the criteria related to compressive strength was also evaluated. According to ASTM C311 [51], the strength activity index of pozzolanic materials must be predicted to know whether the material used as a replacement of cement demonstrates the acceptable level of strength development or not. In ASTM C618, the strength activity index is set as 75%, which means that the cement replacing materials must have compressive strength equal to 75% of CM at seven and 28 days.

The current results show that all of the mortar mixes (Table 5) containing GS, QD, FA and their combinations attained compressive strength more than 75% of the corresponding CM regardless of the percentage replacement of waste materials (GS, QD and FA), their fineness level, age and curing conditions.

### 3.2. Compressive Strength

As described in the Materials section, the effect of GS and QD as a replacement of cement on compressive strength development of mortar was investigated according to ASTM C109. Table 5 shows the overall compressive strength results of this study according to aging, curing moisture and curing temperatures, the type of cement replacing materials used (GS, QD, FA), their different replacement levels (10–20%), their replacement in combinations (GS45 + QD and GS25 + FA) and the fineness of GS (passing 45 and 25 microns). The purpose of using FA was to compare the results of mortar containing local waste (GS and QD) with the corresponding results of mortar containing a well-known pozzolanic material. The different fineness of GS (GS45 passing a 45-micron sieve and GS25 passing a 25-micron sieve) was used to investigate its effectiveness on compressive strength development. The effect of ternary mortar mixes up to 20% replacement with cement (FA10 + GS25-10 and QD10 + GS45-10) was also investigated to find the best possible cement replacement combination. A detailed discussion of the results according to different experimental variables is presented in the following sections.

#### 3.2.1. Effect of Percentage Replacement of Cement with GS, QD and FA

**Figure 6:**
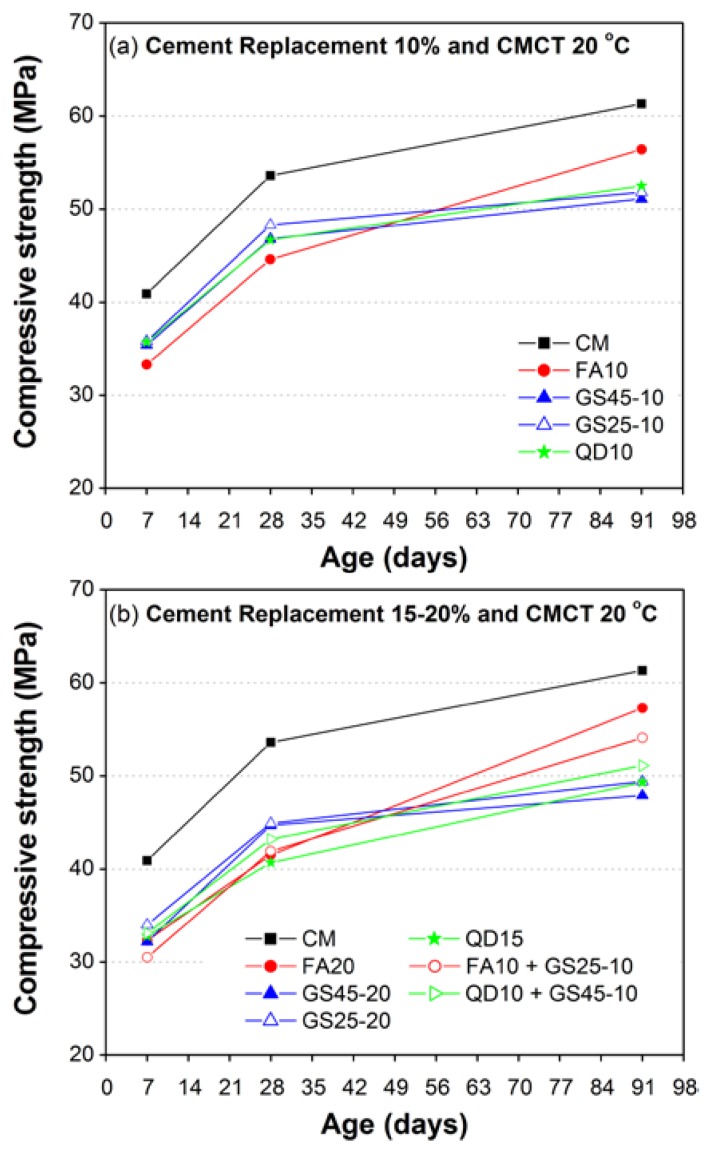
Comparison of the compressive strength development (moist cured under standard curing temperature 20 °C) between CM and mortar containing GS, QD and FA as a partial replacement of cement: (**a**) 10%; (**b**) 15–20%. CMCT, continuously moist cured under a standard curing temperature.

Figure 6 shows the comparison of the compressive strength results of mortars with respect to aging (7, 28 and 91 days) and different percentage replacements of cement with GS (10% and 20% of two different fineness), QD (10% and 15%) and FA (10% and 20%). According to the results of the most recent studies, QD replacement in this study was limited to only 15%, as a higher percentage replacement of cement with QD causes a significant reduction of compressive strength [31]. All of the specimens were continuously moist cured under a standard curing temperature (CMCT) of 20 °C until the age of testing.

As shown in Figure 6, the strength of mortar containing cement replacing materials remained lower than the compressive strength of CM (100% cement) regardless of the type of cement replacing materials used, their percentage replacements and the aging. However, Figure 6a shows that the mortars containing 10% GS and 10% QD demonstrated better early age (seven and 28 days) strength as compared to corresponding mortar containing FA, which might be due to the better packing and filling abilities of GS and QD. Similar results were reported in the earlier studies [21,22,23], according to which the limestone QD normally acts as a filler, accelerates the early age hydration and forms a calcium carbo aluminate.

Moreover, the mortars containing 10% GS or 10% QD demonstrated almost similar strength at all ages. All mortar mixes containing FA (10% FA, 20% FA and 10% FA + 10% GS25) demonstrated lowest early (seven and 28 days) and highest later age (91 days) compressive strength as compared to mortars containing GS or QD. The mortars containing GS (regardless of its fineness and the percentage replacement) and QD (regardless of its percentage replacement) demonstrated lower 91-day strength as compared to corresponding mortars containing FA.

Results of 15% cement replacement with QD were found comparable to 20% cement replacement with GS, except at 28 days, which remained low for QD. However, Figure 6b shows that the later age strength of ternary mortar mixes (10% FA + 10% GS25 and 10% QD + 10% GS45) was enhanced as compared to respective binary mortar mixes (20% GS25 and 20% GS45). The later age strength enhancement of FA mortars demonstrated its high pozzolanic nature at later ages as compared to GS and QD. The lesser later age strength of mortars containing GS or QD could be attributed to their relatively less or very slow pozzolanic activity at later ages. The similar trend of lesser later age strength due to the addition of GS was also reported by other researchers [33,34,35].

Overall, the comparison of the results (Figure 6) shows that the strength of mortars decreases with increasing percentage replacement of cement with other materials (GS, QD and FA), regardless of their type and nature (pozzolanic or filler). Particularly, decreases of strength with increasing percentage replacement of cement are more significant at early ages, which is probably due to less or no pozzolanic activity at early ages (FA is more prominent). Moreover, it may also be worth mentioning here that the effect of the fineness of GS was almost insignificant, as compared to the efforts made to achieve the high fineness GS, though a small enhancement in compressive strength with increased fineness was observed at all ages regardless of the percentage replacement levels of GS. Nonetheless, replacing cement with waste materials (10%, 15% and 20%), though leading to strength loss, may still be beneficial due to several other benefits such as economy, safety of environment, prevention of depletion of natural resources, and so on.

#### 3.2.2. Effect of Curing Temperature on Compressive Strength Development

As discussed in the preceding section, utilization of FA, GS and QD as a replacement of cement tends to decrease the strength of cement mortars when subjected to standard moisture and temperature conditions (continuously moist cured under 20 °C). Previous results (Figure 6) indicated that the utilization of GS and QD might not be that beneficial in terms of gaining strength as compared to pure cement mortar or cement concrete. However, the authors have extended the current research and investigated the trends of strength development of mortars under different isothermal curing temperatures (moderately high 40 °C and high 60 °C). This has been done to gain the maximum benefits out of this research by considering the effects of the local hot environmental conditions of Saudi Arabia. Finally, the results were compared to those as obtained earlier for standard curing conditions (Figure 6). It was expected that the curing under hot temperatures might be beneficial as the ability of materials, in general, gets enhanced at early ages.

**Figure 7:**
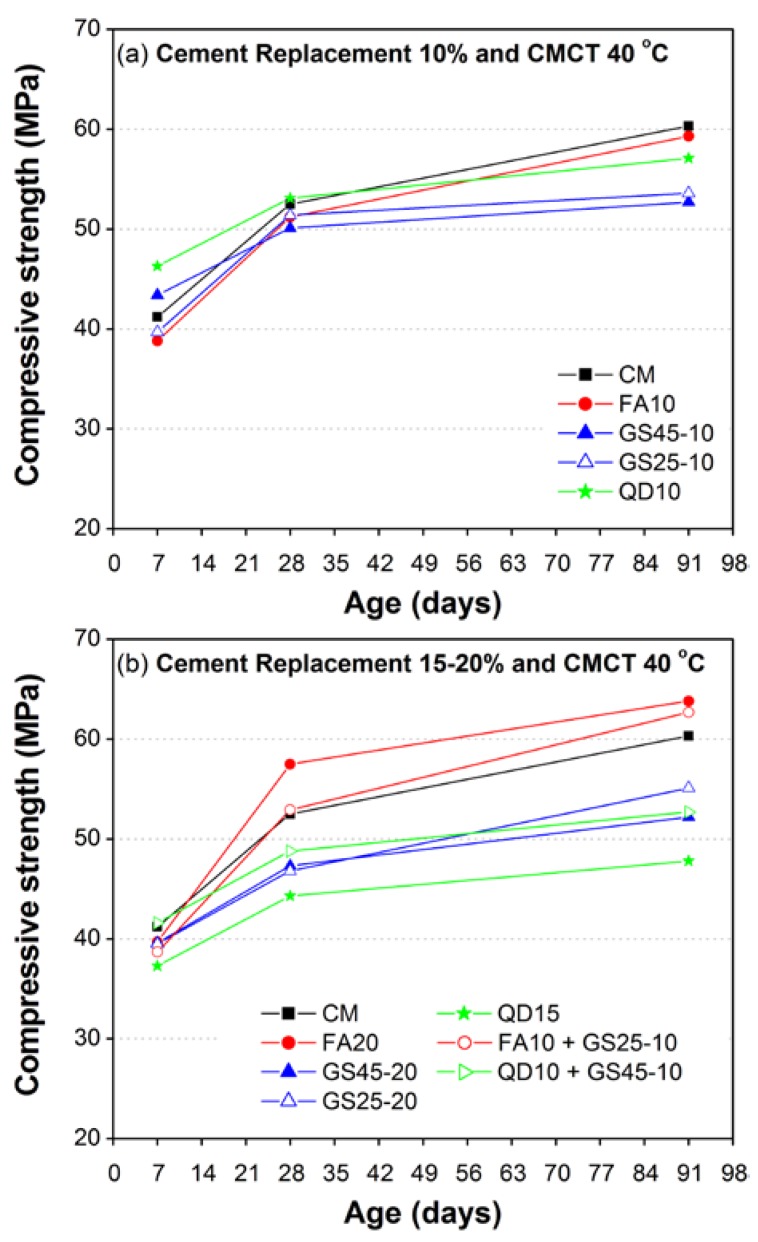
Effect of moderately high curing temperature (40 °C) on compressive strength development of CM and mortar containing GS, QD, and FA as a partial replacement of cement: (**a**) 10%; (**b**) 15–20%.

**Figure 8:**
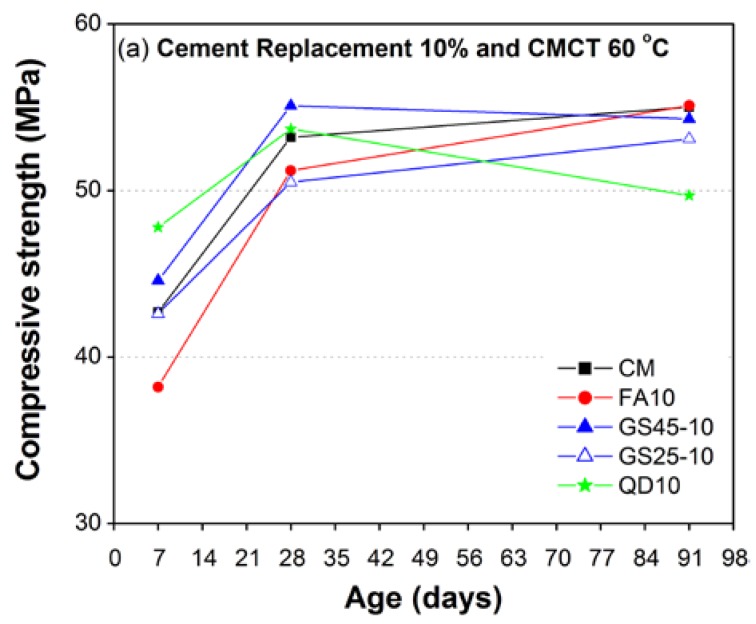

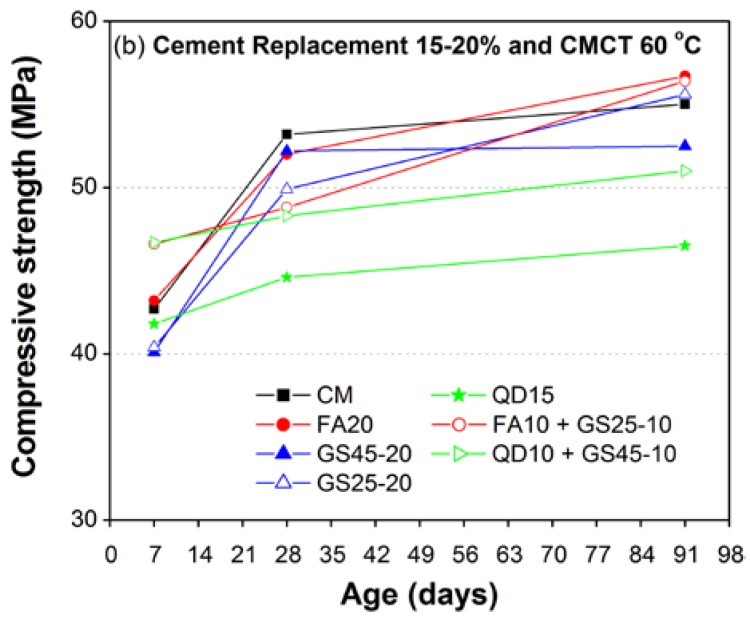
Effect of high curing temperature (60 °C) on compressive strength development of CM and mortar containing GS, QD and FA as a partial replacement of cement: (**a**) 10%; (**b**) 15–20%.

Figure 7 and Figure 8 show the overall comparison of all of the test results of strength development for moderately high (40 °C) and high curing temperatures (60 °C), respectively. Figure 7a and Figure 8a show the test results corresponding to 10% replacement of cement for curing temperatures 40 °C and 60 °C, respectively; while those corresponding to 15–20% are shown in Figure 7b and Figure 8b. The early 7-day compressive strength of mortars containing 10% QD or 10% GS45 (Figure 7a and Figure 8a) was higher than that of the CM mix (cement 100%) both at moderate, as well as high curing temperatures. At later ages (7 and 28 days), the compressive strength of 10% QD decreased with increasing curing temperature and remained the lowest among all materials used in this study.

However, the compressive strength of 10% GS45 decreased at later ages (28 and 91 days) under moderate curing temperature (40 °C) and remained higher at 28 days (and almost same as CM at 91 days) under high curing temperature (60 °C). A reverse trend was found for corresponding replacement levels of FA and GS25 where early 7-day compressive strength was almost the same for GS25 or lower for FA than CM mix both at moderate, as well as high curing temperatures and increased with aging. At 28 days, the compressive strength of both FA and GS25 reached close to CM mix under moderate curing temperature and remained lower under high curing temperature. At 91 days, the compressive strength of FA got better and reached the CM mix both at moderate and high curing temperatures; however, the compressive strength of GS25 remained lower for both temperatures. It is worth noting here that the highest 28-day compressive strength under high curing temperature (60 °C) was developed in 10% GS45, which indicated that the GS passing 45 microns might be a better pozzolanic material to be replaced with cement for casting under high curing temperatures. Even at 91 days, the strength of 10% GS45 was almost similar to CM mix and FA mortar under high curing temperature.

Increasing the percentage replacement with cement such as 15% QD has caused the compressive strength of mortar to be lowered further as compared to 10% QD at all ages both at moderate and high curing temperatures (Figure 7b and Figure 8b). A 20% replacement of cement with GS45 also leads to strength reduction as compared to 10% GS45. However, the effect of increased percentage replacement of cement with GS25 (10–20%) and FA (10–20%) was found insignificant at corresponding curing temperatures. The close or higher compressive strength than CM (early and later age) was found for FA20 and ternary mix (FA10 + GS25-10), both at moderate and high curing temperatures (Figure 7b and Figure 8b). GS25-20 also leads to high later age compressive strength, higher than CM under high curing temperature (60 °C), which was also close to FA20 and ternary mix (FA10 + GS25-10).

As discussed above, the results of this study have clearly demonstrated that the rates of strength development (7–28 and 28–91 days) varied from one material to another under different curing temperatures, which might be due to their different pozzolanic potentials and filling/packing effects. QD might have only a filling effect or very little pozzolanic action at very early ages, as the seven-day compressive strength of 10% QD was the largest and the lowest at 28 and 91 days, both at moderate and high curing temperatures. Unlike QD, GS demonstrated both pozzolanic, as well as filling effects. The rate of strength development (7–28 days) of GS was almost similar to CM mix, and the fastest rate of strength development was observed (7–28 and 28–91 days) in FA10 and FA20, which demonstrated a high pozzolanic potential with increasing curing temperatures. In general, the compressive strength of CM subjected to high temperature at an early age attained higher early age compressive strength, but lower later age compressive strength, and more or less similar trends were observed for mortars containing FA, QD and GS. This phenomenon of high early and low later age strength with increasing curing temperature is referred to as “crossover effect” [52]. In CM and mortar containing FA, a cross-over effect is very clear; however, it is not so obvious in mortars containing GS.

#### 3.2.3. Effect of Fineness of GS with respect to Its Percentage Replacement with Cement, Aging and Curing Temperature

**Figure 9:**
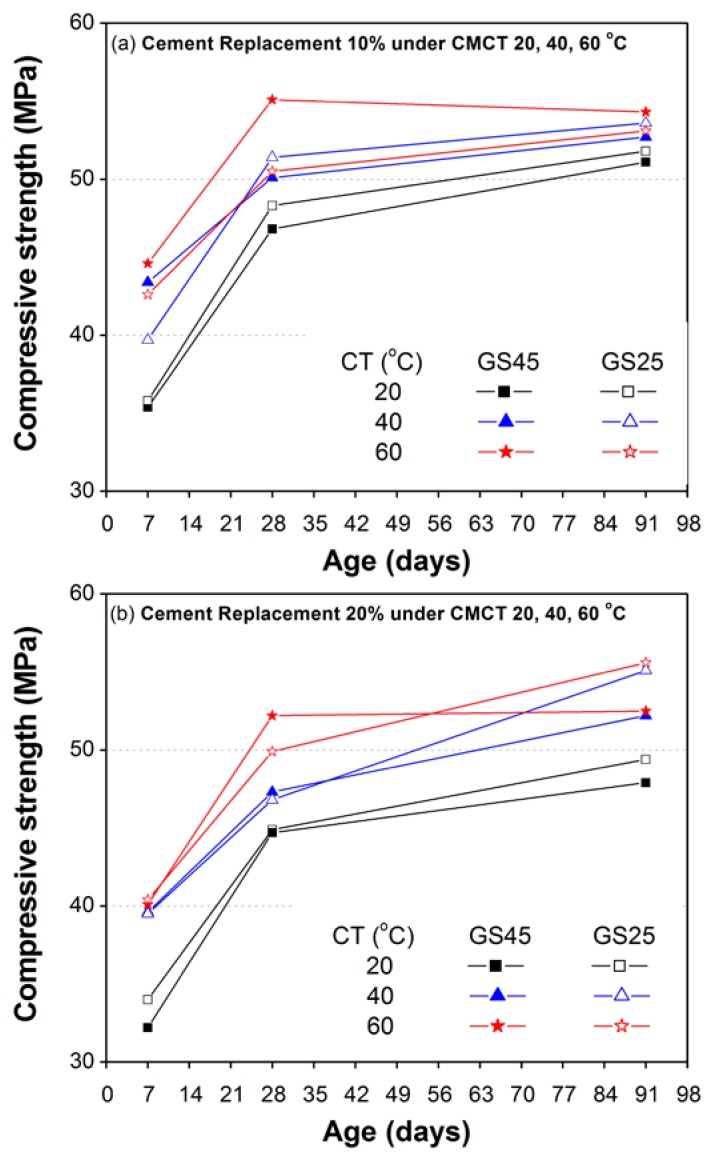
Effect of the fineness of GS on compressive strength of mortar according to aging and curing temperature for: (**a**) 10% replacement with cement; (**b**) 20% replacement with cement.

As discussed earlier, GS having two different fineness levels (Figure 6, Figure 7 and Figure 8) was used to study the influence of its fineness on strength development with respect to percentage replacement, aging and curing temperatures. The method of dry sieving was applied to obtain the GS finer than 45 microns (GS45) and 25 microns (GS25) using micro sieves #325 and #500, respectively. Figure 9 shows the comparison of the test results for the fineness of GS with respect to its percentage replacement with cement, aging and different isothermal curing temperatures.

Figure 9 shows that under normal curing conditions (20 °C continuously moist), GS demonstrated similar strength at early age (7 and 28 days) for both fineness (GS45 and GS25) and percentage replacement with cement (10% and 20%). However, at later ages (91 days), more fineness of GS demonstrated relatively higher strength. A similar trend of strength development was demonstrated when cured under moderately high curing temperature (40 °C), as was observed earlier for normal curing temperature (20 °C), except for 10% replacement as more fineness of GS demonstrated relatively lower strength at seven days. This trend of low early age strength development with increased fineness of GS continued for even a higher curing temperature of 60 °C.

#### 3.2.4. Effect of Continuous and Partial Moist Curing

The effects of two different types of curing on compressive strength development of mortars (CM, FA20 and GS25-20) were also investigated in this study. Identical specimens were cured until the age of testing under two different types of moist curing conditions such as continuous moist curing (CMC) and partial moist curing (first seven days moist (7DM) followed by air curing of relative humidity 60%).

**Figure 10:**
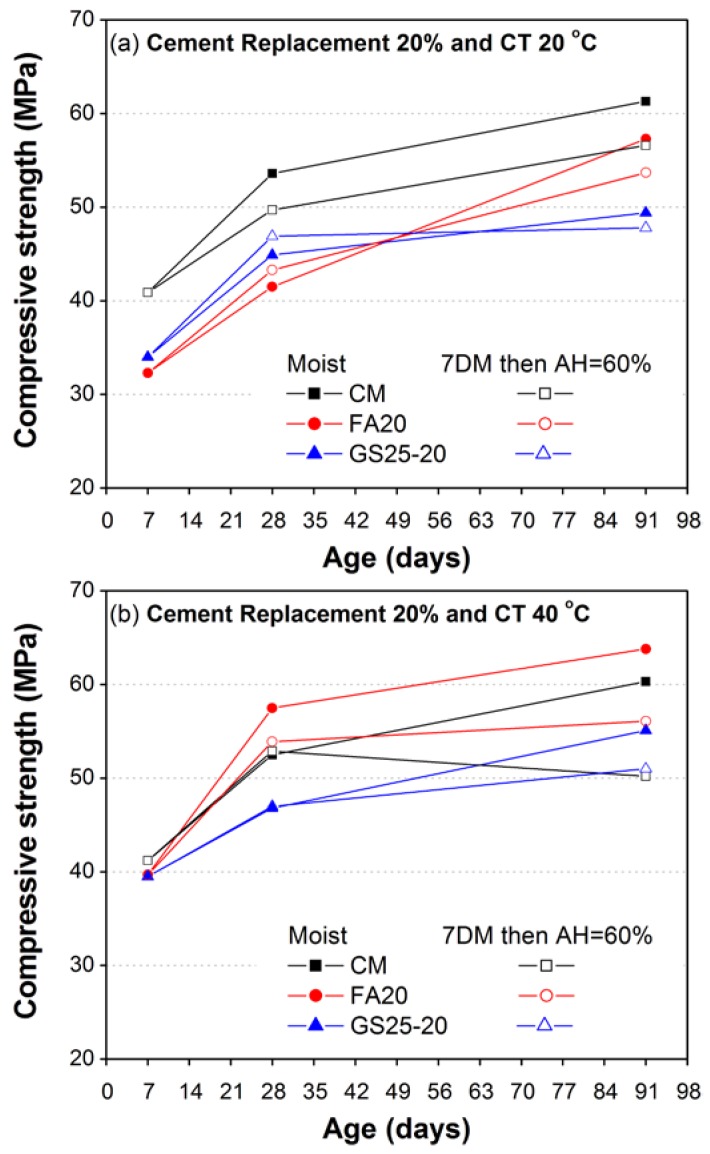

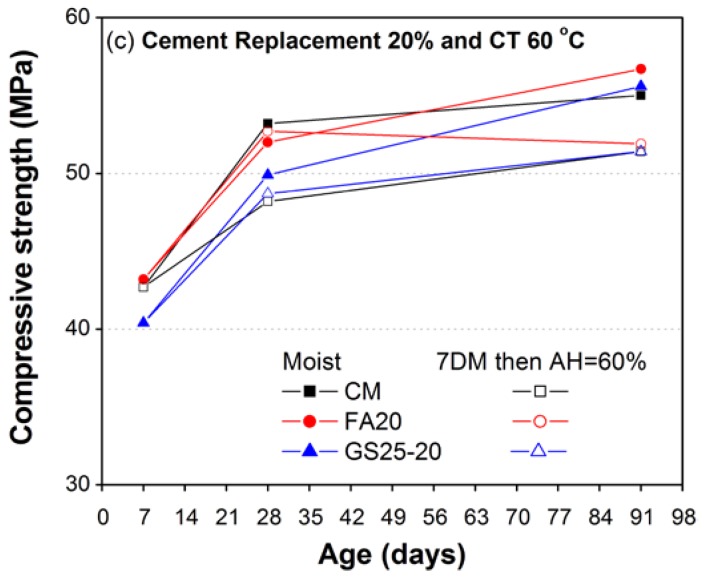
Influence of curing condition (continuous and partial moist curing) on compressive strength of CM and mortar containing 20% FA and 20% GS25 under different curing temperatures: (**a**) 20 °C; (**b**) 40 °C; (**c**) 60 °C.

Both curing conditions were used for three different curing temperatures (20, 40 and 60 °C). Figure 10 shows the comparison of the test results with respect to curing moistures and curing temperatures. As shown in Figure 10a, the compressive strength of CM cured under 20 °C and 7DM remained lower at all ages (28 and 91 days) as compared to identical specimens cured under CMC. However, unlike CM, the compressive strength of mortars containing FA20 and GS25-20 increases at 28 days when cured under 7DM and decreases at 91 days as compared to their corresponding mortars cured under CMC.

Figure 10b,c shows that a similar trend also exists under moderate and high curing temperatures (40 and 60 °C), except that at 28 days, the strength of both mortars FA20 and GS25-20 was almost the same or higher when subjected to CMC as compared to their corresponding mortars cured under 7DM. The strength reduction of mortars at later ages (91 days) under 7DM became more significant under increased curing temperatures (40 and 60 °C), as compared to corresponding mortar cured under a normal temperature of 20 °C. The results (Figure 10) suggest that the CMC of mortar specimens is very beneficial to gain the maximum potential compressive strength of mortars regardless of the curing temperature (20, 40 or 60 °C). The current results (Figure 10c) also suggest a beneficial utilization of GS25-20 under high casting or curing temperature conditions, as the 91-day compressive strength of GS25-20 was almost identical to both CM and FA20, regardless of the type of curing condition.

## 4. Conclusions

This study investigated the compressive strength development of mortars containing different waste materials, such as the lime stone quarry dust (QD) and the industrial granite sludge (GS), as a partial replacement of cement. The influence of different percentage replacements of these materials (GS, QD and FA) with cement, such as 10%, 15%, 20%, fineness of GS (100% passing 45- and 25-micron sieves), curing conditions (moisture and normal (20 °C) and hot environmental temperatures (40 and 60 °C)) were investigated. Finally, the results of this study were compared with those of control mortar (100% cement) and the corresponding mortar containing the fly ash (FA) so as to envisage their potential use in concrete. Based on the current results, the following main conclusions were drawn from this study:

- Under the normal curing conditions of temperature and moisture (20 °C and continuously moist cured), the compressive strength of mortar containing different percentages of waste materials as a substitution of cement (QD, GS, FA and their combinations) remained lower than that of CM at all ages. However, compressive strength slightly increased with increased fineness of GS.
- Unlike standard curing temperature, a significant influence of moderate and high curing temperatures (40 and 60 °C) was observed on the strength development of mortars containing GS. Specifically, the compressive strength of mortar containing 10% GS was comparable to CM or corresponding mortar containing FA up to 28 days and at all ages under moderately high (40 °C) and high curing temperatures (60 °C), respectively. This could be attributed to its increased hydration and pozzolanic reaction at early and later ages, respectively. Moreover, the compressive strength of mortar containing 20% GS was also found comparable to CM and corresponding FA mortar under high curing temperature (60 °C). A combination of 10% GS25 and 10% FA demonstrated comparable strength to CM and corresponding mortar containing FA. Consequently, the current results suggest that high fineness GS should be preferred to achieve better results in terms of strength, especially under high curing temperatures. It is recommended that the greater fineness of GS be achieved to investigate the influence of its high percentage replacement with cement in mortar, as well as in concrete incorporating local environmental conditions.
- The influence of moist and partially moist cuing was significant, as the compressive strength of CM and the mortars containing GS and FA with aging was found lower when subjected to partial moist curing (7DM) as compared to identical specimens subjected to continuous moist curing (CMC). Only at 28 days, the compressive strength of mortars containing FA20 and GS25-20 was higher under 7DM as compared to corresponding mortars cured under CMC. The reduction of strength under 7DM became more significant at later ages (91 days) under moderate and high curing temperatures (40 and 60 °C), as compared to corresponding mortar cured under a normal temperature of 20 °C.
- The results suggest that the CMC should be preferred, as it can be more beneficial in terms of gaining the maximum potential compressive strength of mortars regardless of the type of curing temperature (20, 40 or 60 °C). Specifically, under high casting or curing temperature conditions, the use of GS25-20 can be beneficial, as its 91 day compressive strength was almost identical to both CM and FA20, regardless of the type of curing condition.

## Figures and Tables

**Table 1:** Grain size distribution of fine aggregate (EN 196-1 and ISO 679:2009).

Sieve #	Sieve Size (mm)	Weight Retained (g)	Weight Retained (%)	Cumulative Passing (%)	Cumulative Retained (%)
3/8 inch	9.5	0	0	100	0
No. 4	4.75	0	0	100	0
No. 8	2.36	0	0	100	0
No. 16	1.18	134	26.8	73.2	26.8
No. 30	0.600	179	35.8	37.4	62.6
No. 50	0.300	49	9.8	27.6	72.4
No. 100	0.150	98.8	19.76	7.84	92.16
Pan	-	39.2	7.84	0	-
Fineness Modulus (FM) = (0 + 0 + 0 + 26.8 + 62.6 + 72.4 + 92.16)/100 = 2.54					

**Table 2:** Physical and chemical analyses of materials. FA, fly ash; GS, granite sludge; QD, quarry dust.

**Item**	**C**	**FA**	**GS**	**QD**
	Physical properties			
Specific gravity (g/cm^3^)	3.15	2.83	2.50	2.43
Fineness (m^2^/kg) (Blain)	344	-	-	-
Fineness (m^2^/cc) by Microtrac S3500	0.5670	1.027	1.882 (GS45) 2.047 (GS25)	0.1800
	Chemical properties (oxides, % by weight)			
SiO_2_	20.9	51.5	62.1	5.05
Al_2_O_3_	5.18	24.3	12.4	0.45
Fe_2_O_3_	3.04	8.87	9.80	0.18
(SiO_2_ + Al_2_O_3_ + Fe_2_O_3_) *	-	84.7	84.3	-
CaO	63.9	5.15	4.50	49.8
MgO	1.65	3.50	0.59	-
Na_2_O	0.10	2.38	3.30	-
K_2_O	0.52	1.47	4.40	-
SO_3_	2.61	0.23	0.10	-
LOI **	2.51	0.25	2.71	44.6
	Compounds (%)			
C_2_S	52.1	-	-	-
C_3_S	19.6	-	-	-
C_3_A	8.17	-	-	-
C_4_AF	8.81	-	-	-

* ASTM C618. ** Loss on ignition.

**Table 3:** Particle size distribution of cement, FA, GS45, GS25 and QD.

Materials	Mean (μm)	Standard Deviation (μm)	d_10_ (μm)	d_50_ (μm)	d_90_ (μm)
Cement	10.58	10.01	0.954	4.440	28.63
FA	5.840	4.000	0.694	1.819	13.59
GS45	3.190	1.623	0.633	1.249	7.770
GS25	2.932	1.823	0.563	0.985	7.350
QD	33.35	28.98	4.390	16.65	91.68

**Table 4:** Mixture proportions of mortar (w/cementitious materials (cm) = 0.485; cm:s = 1:2.75).

Batch Quantities (g) for Nine 50-mm^3^ Mortar Specimens							
Mix ID	Water (w)	Cement (c)	GS45	GS25	QD	FA	Sand (s)
Control Mortar (CM)	364	750	0	0	0	0	2063
10% FA (FA10)	364	675	0	0	0	75	2063
20% FA (FA20)	364	600	0	0	0	150	2063
10% GS passing sieve 45 μ (GS45-10)	364	675	75	0	0	0	2063
20% GS passing sieve 45 μ (GS45-20)	364	600	150	0	0	0	2063
10% GS passing sieve 45 μ (GS25-10)	364	675	0	75	0	0	2063
20% GS passing sieve 45 μ (GS25-20)	364	600	0	150	0	0	2063
10% QD (QD10)	364	675	0	0	75	0	2063
15% QD (QD15)	364	638	0	0	112	0	2063
FA10 + GS25-10	364	600	0	75	0	75	2063
QD10 + GS45-10	364	600	75	0	75	0	2063

**Table 5:** Compressive strength (MPa) of mortars with respect to aging, curing temperature and moisture. CM, control mortar.

Mix ID	Curing Moisture	Curing Temperature (°C)								
		20			40			60		
		Age (Days)								
		7	28	91	7	28	91	7	28	91
CM	M *	40.9	53.6	61.3	41.2	52.5	60.3	42.7	53.2	55.0
		(0.9) ***	(1.9)	(3.1)	(1.4)	(2.4)	(6.6)	(2.6)	(1.2)	(1.6)
	7DM **		49.7	56.6		52.9	50.2		48.2	51.4
			(4.2)	(1.6)		(3.2)	(1.9)		(5.3)	(2.3)
FA10	M	33.3	44.6	56.4	38.8	51.2	59.3	38.2	51.2	55.1
		(0.7)	(2.1)	(0.8)	(3.1)	(6.2)	(3.7)	(1.5)	(2.1)	(1.2)
FA20	M	32.3	41.5	57.3	39.7	57.5	63.8	43.2	52.0	56.7
		(2.0)	(2.8)	(4.7)	(2.0)	(5.2)	(2.5)	(2.8)	(2.4)	(1.6)
	7DM		43.3	53.7		53.9	56.1		52.7	51.9
			(1.2)	(2.6)		(0.7)	(0.2)		(3.1)	(5.4)
GS45-10	M	35.4	46.8	51.1	43.4	50.1	52.7	44.6	55.1	54.3
		(1.2)	(0.7)	(1.9)	(1.0)	(1.7)	(4.9)	(2.0)	(1.7)	(2.4)
GS45-20	M	32.2	44.7	47.9	39.6	47.3	52.2	40.1	52.2	52.5
		(0.5)	(1.9)	(2.2)	(1.0)	(5.0)	(1.1)	(0.8)	(1.6)	(3.0)
GS25-10	M	35.8	48.3	51.8	39.7	51.4	53.6	42.6	50.5	53.1
		(0.5)	(2.3)	(1.4)	(1.4)	(2.6)	(1.8)	(1.9)	(1.7)	(4.1)
GS25-20	M	34.0	44.9	49.4	39.5	46.8	55.1	40.4	49.9	55.6
		(0.4)	(1.9)	(1.6)	(0.3)	(1.5)	(3.2)	(0.6)	(0.8)	(1.5)
	7DM		46.9	47.8		47.0	51.0		48.7	51.4
			(2.3)	(4.6)		(3.7)	(6.8)		(2.3)	(1.8)
QD10	M	35.7	46.7	52.5	43.8	48.7	51.3	44.8	48.7	47.2
		(0.2)	(1.5)	(3.2)	(2.4)	(0.4)	(2.0)	(2.8)	(2.2)	(0.4)
QD15	M	32.8	40.7	49.3	37.3	43.0	47.8	39.3	43.1	42.8
		(1.1)	(2.0)	(3.0)	(0.6)	(1.2)	(1.5)	(1.8)	(1.1)	(1.6)
FA10 + GS25-10	M	30.5	41.9	54.1	38.7	52.9	62.7	46.6	48.8	56.4
		(2.1)	(1.9)	(3.9)	(1.0)	(2.5)	(0.8)	(0.9)	(1.2)	(1.9)
QD10 + GS45-10	M	33.1	43.2	51.1	39.9	48.8	52.7	46.7	48.3	51.0
		(1.0)	(1.8)	(2.3)	(0.1)	(0.6)	(1.0)	(2.5)	(0.9)	(1.3)

* Continuously moist-cured until the age of testing. ** First 7 days moist curing followed by air-curing (60% humidity) until the age of testing. *** Values in parenthesis indicate the standard deviation of all test results.
